# Cardiac Function and Serum Biomarkers throughout Staged Fontan Palliation: A Prospective Observational Study

**DOI:** 10.3390/jcdd10070289

**Published:** 2023-07-07

**Authors:** J. P. G. van der Ven, V P. Kamphuis, E van den Bosch, D Gnanam, C Terol, A J. J. C. Bogers, J. M. P. J. Breur, R. M. F. Berger, N. A. Blom, A. D. J. ten Harkel, L. Koopman, W. A. Helbing

**Affiliations:** 1 Division of Pediatric Cardiology, Department of Pediatrics, Erasmus MC Sophia Children’s Hospital, 3015 CN Rotterdam, The Netherlands; 2 Netherlands Heart Institute, 3501 DG Utrecht, The Netherlands; 3 Department of Cardiothoracic Surgery, Erasmus MC, 3015 CN Rotterdam, The Netherlands; 4 Division of Pediatric Cardiology, Department of Pediatrics, Leiden University Medical Center, 2300 RA Leiden, The Netherlands; 5 Division of Pediatric Cardiology, Department of Pediatrics, University Medical Center Utrecht, 3508 GA Utrecht, The Netherlands; 6 Division of Pediatric Cardiology, Department of Pediatrics, University Medical Center Groningen, 9713 GZ Groningen, The Netherlands; 7 Division of Pediatric Cardiology, Department of Pediatrics, Amsterdam University Medical Center, 1007 MB Amsterdam, The Netherlands

**Keywords:** congenital heart disease, Fontan palliation, single ventricle, biomarkers

## Abstract

Fontan patients undergo multiple cardiothoracic surgeries in childhood. Following these procedures, ventricular function is temporarily decreased, and recovers over months. This is presumably related to cardiopulmonary bypass, but this is incompletely understood. Throughout the Fontan palliation, cardiac function is also affected by volume unloading. We aimed to gain insight into the biological processes related to impaired ventricular function and recovery following Fontan palliations using a panel of biomarkers. Furthermore, we described changes in ventricular function across the Fontan palliation due to volume unloading. We performed a prospective multicenter observational study in patients undergoing partial (PCPC) or total cavo-pulmonary connection (TCPC). Patients underwent assessment—including echocardiography and blood sampling—before surgery (T1), at first follow-up (T2), and 1 year after their procedures (T3). Blood samples were analyzed using a biomarker panel (OLINK CVD-III). Ninety-two biomarkers were expressed as principal components (PC) to limit multiple statistical testing. We included 32 PCPC patients aged 7.2 [5.3–10.3] months, and 28 TCPC patients aged 2.7 [2.2–3.8] years. The single ventricular longitudinal strain (SV GLS) temporarily decreased for PCPC patients at T2 (−15.1 ± 5.6 (T1) to −13.5 ± 5.2 (T2) to −17.3 ± 4.5 (T3), *p* < 0.047 for all differences), but not following TCPC. The serum biomarkers were expressed as 4 PCs. PC1, including biomarkers of cell–cell adhesion, was not related to any patient characteristic. PC2, including biomarkers of superoxide anion regulation, increased at T2. PC3, including biomarkers of cardiovascular development, related to the stage of Fontan palliation. PC4 was of uncertain biological or clinical significance. No PC was found that related to ventricular performance. The SV GLS was temporarily diminished following PCPC, but not following TCPC. Several biomarkers were related to post-operative stress and adaptation to the PCPC or TCPC circulation, but none were related to the outcome.

## 1. Introduction

Children born with univentricular congenital heart disease require a series of extensive cardiothoracic surgeries to palliate their congenital heart defects [1]. The result of these procedures is a Fontan circulation, in which the single ventricle supplies the systemic and pulmonary circulation in series, rather than parallel [1]. Although this palliative strategy results in dramatically improved survival, the lifetime morbidity for these patients remains high [1]. It should be considered that the multitude of cardiothoracic surgeries performed in childhood, although necessary, is not without harm. A higher number of previous interventions may be related to worse short-term outcomes [2], and prolonged cardiopulmonary bypass (CPB) time is considered a risk factor for long-term outcomes, including overall survival [3,4]. Thus, the extent of myocardial injury in childhood may be an important predictor of lifelong success of the Fontan circulation, among other factors such as low pulmonary vascular resistance and good atrioventricular valve function.

The biological processes of myocardial injury in relation to surgery for congenital heart disease (CHD) are poorly understood. Following surgery for CHD ventricular performance may be depressed, but recovers over the course of months [5,6,7]. This temporarily impaired ventricular function is thought to be related to peri-operative conditioning, which includes surgical trauma, mechanical stress, blood reaction with foreign materials, and—importantly—cardiopulmonary bypass [8]. The oxidative stress related to cardiopulmonary bypass is considered central in the pathogenesis [8,9]. Although essential for surgery, CPB exposes to some extent the heart to ischemia and reperfusion injury, to some extent, which may lead to myocardial dysfunction and even cardiomyocyte death [8,9]. Prolonged CPB may be necessary to complete the surgical procedure, but may cause more cardiomyocyte death rather than reversible myocardial dysfunction. Thus, prolonged CPB may relate to worse long-term outcomes. The exact processes by which the myocardium recovers from CPB injury are poorly understood. Characterization of the cardiovascular and immunological response to cardiothoracic surgery may allow for characterization of favorable recovery, provide more insights into the mechanisms of recovery, and may ultimately lead to treatment strategies to minimize myocardial damage.

We performed a prospective observational study of patients undergoing partial cavo-pulmonary connection (PCPC, also referred to as the bidirectional Glenn procedure) or total cavo-pulmonary connection (TCPC, also referred to as the Fontan procedure) surgery. Our aim was to identify changes in cardiovascular serum biomarkers and echocardiographic functional parameters at different time points around the index surgery to gain insight into the biological processes related to temporarily impaired ventricular function and subsequent recovery following Fontan procedures.

## 2. Materials and Methods

We performed a multicenter prospective observational study. The study protocol was registered at the Netherlands Trial Register (NL5129). Patients scheduled to undergo PCPC or TCPC were included, from December 2015 to September 2019, from the participating centers (Erasmus MC Sophia Children’s Hospital, Rotterdam; Willem Alexander Children’s Hospital, Leiden; Wilhelmina Children’s Hospital, Utrecht; and the University Medical Center Groningen Beatrix Children’s Hospital, Groningen). Patients with severe intellectual impairment were excluded from participation. Subjects underwent examination at 3 study time points: before surgery (T1); at first outpatient follow-up, or 2 weeks after surgery when hospitalized (T2); and 1 year after surgery (T3). At each time point, a physical exam was conducted, an echocardiography study was carried out, and a blood sample was obtained. The study protocol was approved by the research ethics committees of the participating centers (MEC-2014-326/NL48188.078.14). All participants’ parents and/or legal guardians provided written informed consent in accordance with Dutch legislature.

The patients underwent transthoracic echocardiography studies according to a study protocol based on current guidelines [10]. This protocol included 2D-gray scale; M-mode; color flow Doppler; pulsed-wave Doppler; continuous wave Doppler; and tissue Doppler (TDI) modalities. All of the studies were performed on a Vivid7 or Vivid E9 cardiac ultrasound system (General Electric Vingmed Ultrasound, Horten, Norway) by experienced cardiac sonographers. No patients were sedated for the echocardiography studies. To limit variability across the centers, all postprocessing was performed in 2 core centers (Erasmus MC and Leiden University Medical Center) using commercially available software (EchoPac version 11.2, General Electric Vingmed, Horten, Norway). All of the M-mode and pulsed wave tissue Doppler measurements were averaged over 3 consecutive heart beats. The myocardial strain was obtained from 2D gray-scale images with a frame rate of 60–90 frames per second, using speckle tracking over 1 cardiac cycle. The global longitudinal strain of the single ventricle (SV GLS) was averaged over the available segments of the apical 4-chamber view. In the presence of a ventricular septal defect (VSD), the GLS was assessed from the lateral wall and interventricular septum if the VSD length was smaller than 50% of the septum length in the 4-chamber view, and segments including the VSD were excluded. If the VSD comprised ≥50% of the interventricular septum, the GLS was obtained from the lateral wall segments of the dominant ventricle only. The longitudinal strain of the lateral wall was also reported separately for all patients. S’, E’, and A’ were derived by TDI from the lateral AV valve. For echocardiography parameters, the AV valve of the dominant ventricle was considered when both valves where present.

Venous or capillary blood samples were obtained in EDTA containers. The samples were centrifuged and stored at −80 °C. Blood samples were analyzed using a protein biomarker of a panel of 92 cardiovascular novel biomarkers (OLINK Cardiovascular panel III, Olink Bioscience, Uppsala, Sweden). This panel is analyzed using a proximal extension array [11]. In short, the biomarkers of interest are bound to nucleotide markers, which are subsequently amplified by a polymerase chain reaction (PCR). The concentrations are expressed relative to other samples within the panel as normalized protein expressions (NPXs). NPX is a logarithmic scale, where a 1 unit NPX increase relates to a doubling in protein concentration. For analytes with concentrations under the limit of detection, the reported values were used for analysis (in consultation with OLINK Bioscience). Biomarkers for which the limit of detection was not reached in over 50% of samples were excluded from analyses. The N-terminal pro-brain natriuretic peptide (NT-proBNP) was also analyzed in the clinical laboratories of the participating centers for quality assessment of the panel biomarker analysis. NT-proBNP levels are compared to age- and sex-related reference values [12].

The data are presented as “mean ± standard deviation” for normally distributed data, and “median [interquartile range]” for non-normally distributed data. Categorical data are presented as “count (percentage)”. The data were compared between groups using Student’s *t*-test and the Wilcoxon test, depending on the distribution. Paired tests were used for the comparisons between time points. Panel biomarkers were analyzed using principal components analysis (PCA) [13]. This statistical technique reduces the number of parameters to be analyzed from 92 biomarkers to a few principal components, thereby limiting the false detection rate associated with a large number of statistical tests. PCA summarizes the information contained in a set of possibly correlated biomarkers as uncorrelated principal components. PCA was performed on a dataset of panel biomarker expressions only, with each blood sample as an individual entry. The principal component scores summarized the serum levels of a group of correlated biomarkers as a single dimensionless value. All principal components that explained ≥5% of variance in the dataset (i.e., information contained in the dataset) were considered for analysis. Statistical analyses regarding principal component scores were repeated for the top 5 individual biomarkers contributing to that principal component score. Biological functions associated with principal components were determined by protein enrichment analysis (STRING version 11.5) [14]. The intra-observer and inter-observer variabilities of speckle tracking echocardiography parameters were assessed in a subset of 15 randomly selected echocardiography studies. All statistical analyses was performed in R (R Foundation for Statistical Computing, Vienna, Austria). A *p* value < 0.05 was considered statistically significant. No corrections for multiple statistical testing were performed, as PCA reduced the number of parameters to be analyzed to an amount that is conventional in biomedical research.

## 3. Results

### 3.1. Study Cohort

We included 32 patients who underwent PCPC and 28 who underwent TCPC. There were 8 patients who participated in the study for both PCPC and TCPC palliations, thus 52 unique patients were included. Patient characteristics are presented in Table 1. The patients who underwent PCPC and TCPC were comparable with regard to (extra)cardiac defects. All of the patients underwent interventions as part of a staged Fontan palliation. Concomitant procedures included correction of total anomalous pulmonary venous return during PCPC (*n* = 1), mitral valve plasty during TCPC (*n* = 1), and tricuspid valve plasty during TCPC (*n* = 2). Of the TCPC patients, 15 (54%) underwent extra-cardiac conduit (ECC) TCPC and 13 (46%) underwent intra-atrial lateral tunnel (ILT) repair. Two ECC patients underwent off-pump procedures. Furthermore, 5 ECC patients had a fenestrated TCPC. Assessments took place 1 [1–4] day before intervention (T1), 10 [7–15] days after surgery (T2), and 358 [301–458] days after surgery (T3). Twenty-two patients had complications following surgery. Two patients died due to circulatory failure following PCPC (no patients died following TCPC); one patient underwent PCPC take-down the same day because of persistently high superior caval vein pressure. These complications occurred before the T2 assessment took place. As such, only T1 measurements were included in the analysis for these patients. Furthermore, 2 PCPC patients underwent surgical re-intervention for bleeding on the same day and 1 day post-operatively, respectively; one patient underwent unplanned stenting of the aorta and coiling of major aorta-pulmonary collateral arteries 4 days after PCPC. Other complications included chylothorax (*n* = 9 TCPC); infection (*n* = 2 PCPC; *n* = 3 TCPC; 2 pneumonia, 3 upper respiratory infections, 1 with concomitant gastro-enteritis); post-pericardiotomy syndrome (*n* = 1 PCPC); and seizure (*n* = 1 PCPC). These complications did not impede the study assessments. There was no significant difference in the mean arterial pressures for PCPC (73 [67–80] versus 70 [65–75] versus 69 [64–77] mmHg, *p* > 0.248 for all differences) or TCPC (72 [66–86] versus 77 [67–83] versus 77 [71–85] mmHg, *p* > 0.305 for all differences) patients.

### 3.2. Echocardiography

Echocardiography studies were obtained at T1 (*n* = 50), T2 (*n* = 51), and T3 (*n* = 51). Parameters of the echocardiography studies are presented in Table 2. For PCPC patients, SV GLS developed from −15.1 ± 5.6% at T1, to −13.5 ± 5.2% at T2, to −17.3 ± 4.5% at T3 (*p* = 0.047 for T1 to T2, *p* = 0.016 for T2 to T3). Other parameters of ventricular performance (lateral wall LS, TDI AV S’ and AV-APSE) followed similar patterns to SV GLS. The subjective contractility decreased from good to moderate for 4 PCPC patients between T1 and T2. Additionally, 1 PCPC patient’s contractility decreased from good to poor between T1 and T2. Conversely, for only 1 PCPC patient, the contractility increased from moderate to good from T1 to T2. For all of the TCPC patients, the subjective contractility was similar at T1 and T2. PCPC patients with a dominant RV had lower SV GLS at T2 compared to patients with a dominant LV (−11.4 ± 4.6% versus −16.5 ± 4.4%, *p* = 0.023), although the SV GLS was similar between these groups at T1 and T3. E/A and E/E’ did not differ across the study time points for PCPC patients. For TCPC patients, SV GLS—or any other parameter of ventricular performance—did not differ across the study time points. No differences in the SV GLS between patients with a left and right dominant ventricle were found. The SV GLS was borderline more favorable for ILT patients compared to ECC patients at T3 (SV GLS −20 ± 2% versus −17 ± 3%, *p* = 0.089; TDI AV S’ 5.8 ± 1.1 versus 4.8 ± 1.0 cm/s, *p* = 0.064; AV-APSE: 11 ± 2 versus 7 ± 2 mm, *p* = 0.035). E/A and E/E’ did not differ across the study time points for TCPC patients. At T1, moderate to severe regurgitation of any AV valve was present for 7 PCPC and 5 TCPC patients, respectively. No patients had moderate to severe regurgitation of the (neo)aortic valve at T1. At T2, 5 PCPC patients had moderate to severe regurgitation of any AV valve, and additionally 1 PCPC patient had moderate to severe regurgitation of the (neo)aortic valve. At T3, 2 PCPC patients had moderate to severe regurgitation of any AV valve. No patients had moderate to severe regurgitation of the (neo)aortic valve at T3. The SV GLS did not differ between patients with and without a fenestration at any time point. The SV GLS did not differ between patients with and without moderate to severe ventriculo-arterial or AV valve regurgitation (combined for PCPC and TCPC patients) at any study time point.

### 3.3. Serum Biomarkers

**Figure 1:**
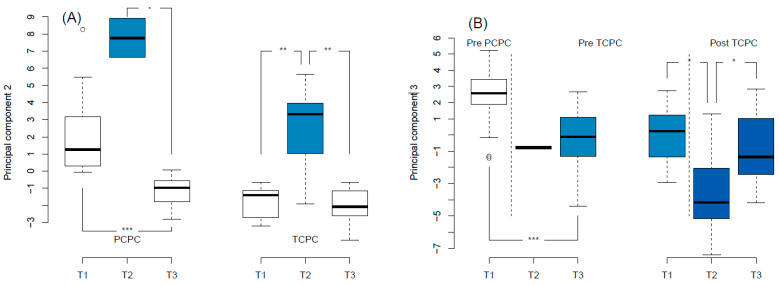
Principal component analysis. (**A**): Principal component 2 scores increased at the early post-operative study time point (T2). (**B**): Principal component 3 scores differed across Fontan palliation stages. Colors are arbitrary and added for clarity. PCPC: partial cavo-pulmonary connection; TCPC: total cavo-pulmonary connection. * *p* < 0.05; ** *p* < 0.01; *** *p* < 0.001.

Serum panel biomarkers were obtained for 31 patients at T1, 9 patients at T2, and 32 at T3. One biomarker (Spondin-1) was expressed lower than the limit of detection in all subjects, and was excluded from analysis. One sample had an unusually low expression of all biomarkers (average NPX Z = −9.3, compared to other samples), and was excluded as an outlier. In total, 91 biomarkers were analyzed in 71 samples from 33 patients. NT-proBNP measurements from the biomarker panel correlated with (log-transformed) measurements obtained in clinical laboratories (r = 0.82, *p* =< 0.001). The 91 biomarkers were reduced to 4 principal components, which together explained 58% of the total variance in the dataset. Principal components information, including highly contributing biomarkers and biological functions, are summarized in Table 3. Additional analyses for individual biomarkers contributing to specific principal components are presented in the Supplementary Materials. The largest principal component (PC1)—accounting for 37% of variance in the dataset—did not relate to any study parameter. The biomarkers that contributed to this principal component included urokinase receptor (U-PAR), tyrosine-protein kinase receptor UFO (AXL), and intercellular adhesion molecule 2 (ICAM2). PC2 was primarily associated with early post-operative status (T2), as shown in Figure 1A. The same was true for individual biomarkers that contributed to this PC (Table 4). Furthermore, PC2 increased at the pre-PCPC stage, compared to the 1 year follow-up (2.0 ± 2.2 versus −1.0 ± 0.8, *p* < 0.001). Principal component 3 was primarily associated with the stage of Fontan palliation (Figure 1B). Of the contributing individual biomarkers, perlecan (ANOVA test *p* = 0.003) and collagen type 1 alpha 1 chain (ANOVA test *p* =< 0.001) differed across stages of the Fontan palliation. No principal component related to single ventricular GLS at any study time point. The pre-operative PC3 scores differed between TCPC patients who developed complications versus those who did not (−1.0 ± 1.2 versus 1.1 ± 1.4, *p* = 0.018). For the contributing biomarkers, this was only true for collagen type 1 alpha 1 chain (3.9 ± 0.2 versus 3.6 ± 0.1, *p* = 0.015). Higher pre-operative PC4 scores correlated with a longer post-operative intensive care unit (ICU) stay duration for patients undergoing PCPC (rho = 0.59, *p* = 0.010), although this was not true for any individual biomarker contributing to PC4. For patients undergoing TCPC, higher pre-operative PC1 scores correlated with a shorter ICU stay (rho = −0.58, *p* = 0.037). Of the individual contributing biomarkers, only higher tumor necrosis factor receptor type 1 related to a longer ICU stay. No principal component related to intervention duration, perfusion duration, or aortic cross-clamp duration. Furthermore, no principal component scores differed between patients with a left versus right dominant ventricle, cardiac diagnosis, sex, or between ECC and ILT modifications (for TCPC patients).

The NT-proBNP levels, assessed using standard clinical laboratory testing, were higher at T1 for the PCPC patients compared to the TCPC patients (102 [36–124] versus 23 [11–33] pmol/L, *p* < 0.001). At this stage, NT-proBNP was above age-related references for 1 TCPC patient. From T1 to T2, NT-proBNP did not increase for PCPC patients, but did increase for TCPC patients (23 [11–33] versus 49 [26–126] pmol/L, *p* < 0.001). At T2, NTpro-BNP increased for 2 TCPC patients compared to reference values. At T3, NTpro-BNP increased for 1 PCPC patient compared to the reference values. The log-transformed NT-proBNP assessed by clinical laboratory testing correlated excellently with the NT-proBNP assessed via panel biomarker analysis (r = 0.82, *p* < 0.001).

## 4. Discussion

In this multicenter prospective observational study, we found temporarily decreased ventricular function following PCPC. In our patients who underwent TCPC, we did not note these changes in ventricular function. We used a panel of cardiovascular biomarkers to characterize the response to interventions, and identified biomarker phenotypes (i.e., principal components in the biomarker data) that were related to the recovery and stages of Fontan palliation. No biomarker phenotype related to impaired ventricular function following palliation for both PCPC and TCPC surgery.

The single-ventricle GLS for Fontan patients was impaired at each study time point compared to published pediatric reference values for left or right ventricular GLS [15]. We found that the SV GLS decreased shortly following PCPC, but returned to a level above pre-operative levels at the 1 year follow-up. This temporarily impaired ventricular performance is a known phenomenon that has previously been reported for several CHD diagnoses [16]. Recently, Vincenti et al. reported that ventricular impairment—assessed by ejection fraction obtained during cardiac catheterization—at hospital discharge and up to the months following PCPC was more severe for patients with a dominant right ventricle compared to those with a dominant left ventricle [17]. Our present study confirms this finding. Importantly, Vincenti et al. found patients with more severely impaired ventricular performance after PCPC had worse prognoses during a median 8 year follow-up [17]. In this study by Vicenti et al., ventricular performance recovered to baseline levels or above in the following 8–12 months, except for patients with hypoplastic left heart syndrome. Aortic cross-clamping is an important determinant of peri-operative injury. Despite applying cross-clamping of the aorta, and thus having aortic cross-clamp time for TCPC as compared to PCPC (where aortic cross-clamping is uncommon), we did not find temporarily impaired ventricular performance following TCPC palliation. This is in concordance with previous studies [18,19,20]. Tham et al. found, in a prospective study, that longitudinal and circumferential strain of the systemic right ventricle did not differ before versus after TCPC completion in 76 patients with hypoplastic left heart syndrome [18]. In retrospective studies, no difference in the RV GLS and RV global circumferential strain [19] or tissue Doppler myocardial performance index [20] was found before versus shortly after TCPC surgery. It should be noted that most measures of ventricular performance (i.e., SV GLS, TDI AV S’, and AV-APSE) are dependent on ventricular loading conditions, and furthermore will change with normal somatic growth [15]. Important volume unloading occurs throughout the Fontan palliation, which affects these markers of ventricular function. The volume unloading of the single ventricle following PCPC may have more impact on echocardiographic parameters compared to the changes surrounding TCPC [21,22].

We found that NT-proBNP levels generally decrease throughout Fontan palliation. These results are consistent with previous studies, and probably relate to volume unloading throughout the surgical stages, although normal physiological NT-proBNP levels also decrease throughout childhood [12,23,24]. A transient increase was seen following TCPC surgery, probably relating to impaired ventricular function. Previous studies found pre-operative NT-proBNP may have value as a marker for risk in congenital heart disease [25,26]. 

Our panel biomarker analysis identified several phenotypes. The first principal component—accounting for the largest variance in the dataset—surprisingly did not relate to a clear clinical phenotype. The biological functions associated with these biomarkers were related to—as determined by protein enrichment analysis—components of the cell surface and the plasma membrane. The high variance along this principal component may be explained by confounding factors not considered in the current study, as discussed in the limitations section.

The second principal component primarily related to early post-operative status for both PCPC and TCPC procedures (i.e., the second operative week). Furthermore, PC2 scores increased at the PCPC pre-operative time point compared to other study time points, including for that of the TCPC. This may relate to the degree of cyanosis at different time points, as some biomarkers contributing to this PC may be influenced by cyanosis [27,28]. The scores of this principal component were primarily determined by the expressions of integrin beta 1 (ITGB1), growth/differentiation factor 15 (GDF-15), and epidermal growth factor receptor (EGFR). These biomarkers have many functions in different organ systems and disease states, although the regulation of superoxide anion generation is a common biological process across these biomarkers. During and following cardiopulmonary bypass, the myocardium is exposed to a large burden of oxidative stress [8,16]. This may damage proteins related to excitation and contraction, and is considered a central feature in the pathophysiology of temporarily impaired ventricular function following cardiothoracic surgery [8]. Principal component 2, although related to the early post-operative time point, did not relate to early post-operative ventricular function in our current study. This may relate to the limited number of blood samples obtained at T2. Biomarkers contributing to this PC, i.e., ITGB1 [29], GDF-15 [30], and EGFR [31], have been linked to favorable recovery from cardiac injury. In the peri-operative setting, these biomarkers probably reflect an immune response to injury, and may ameliorate the associated myocardial damage. Recently, GDF-15 was found to be related to exercise capacity [32], as well as clinical outcome [32,33], for adolescent Fontan patients.

Principal component 3 related to the stage of Fontan palliation. Patients before PCPC palliation had the highest principal component 3 scores, and patients following TCPC had the lowest scores. The biomarkers that contributed to principal component 3—among which perlecan (PLC), myoglobin (MB), and collagen alpha-1(I) chain (COL1A1)—relate to extracellular matrix organization, blood vessel development, and circulatory system development. COL1A1 is of particular interest, as this individual biomarker both differed in expression across stages of Fontan palliation, and was pre-operatively lower for patients who developed post-operative complications. In general, this principal component may reflect the adaptation to post-PCPC or post-TCPC circulation. This could include re-organization of the extracellular matrix of the vascular system, but may also include musculoskeletal adaptations, which can be abnormal in Fontan patients [34]. It is currently uncertain whether the biomarker response across Fontan stages reflects favorable or unfavorable adaptation.

Principal component 4 was of unknown clinical significance. The biomarkers highly contributing to this principal component mostly related to leukocyte and platelet function. Leukocyte activation is an important aspect of the immune response to cardiopulmonary bypass in children [35,36]. However, in our present study, principal component 4 did not relate to the post-operative time point. Furthermore, it is unclear whether this PC relates to outcomes.

Recent proteomic and metabolomics studies found differences between univentricular CHD patients before versus after PCPC [37], and between pediatric Fontan patients with versus without heart failure [38]. Biomarkers related to cardiovascular, metabolic, and immunologic function may provide early signs of circulatory failure. More comprehensive characterizations of these processes may be used to identify patients at risk for adverse outcomes, and provide insight into the biological processes governing the response to peri-operative injury and cardiovascular adaptation to post-PCPC and post-TCPC circulation. Increasing our understanding of these biological processes could aid in identifying potential targets for therapy, which may include myocardial protective strategies or inhibition of disadvantageous immune responses.

### Limitations

Despite this study’s strengths, such as the prospective and multicenter study design, some limitations should be considered. Our study population was relatively small and heterogeneous. Differences in cardiac diagnosis and extracardiac comorbidities, including genetic defects and heterotaxy syndrome, may be confounders for our analyses. 

The echocardiographic parameters of ventricular performance used in this study are dependent on ventricular loading conditions. PCPC and TCPC procedures reduce ventricular filling, and thus preload. Parameters of single ventricular preload, such as cardiac magnetic resonance imaging-derived end diastolic volumes or invasive measures of ventricular filling pressures, were not available in this study. Therefore, it remains uncertain whether the decrease in echocardiographic parameters of contractility reflect true dysfunction.

The panel approach to biomarker analysis used in this study should be considered as an exploratory approach. The proximal extension array results in relative concentrations rather than exact quantifications. Therefore, no comparison to published references values can be made. Furthermore, the biological functions and behavior of biomarkers may be poorly defined. Confounding factors, such as variations related to the time of day (i.e., diurnal rhythms) [39,40,41], postprandial status [42], environmental factors, (epi)genetic differences, or yet unknown factors, may affect circulating biomarker levels. We assessed a multitude of biomarkers using principal component analysis. This strategy reduced the number of parameters to be assessed from 91 biomarkers to 4 principal components. It should be noted that these principal components accounted for only 58% of the variance (i.e., information) in the dataset. Additional principal components would each explain less than 5% of the total variance, and were deemed unlikely to be clinically relevant. Considering a multitude of additional principal components would increase the statistical risk of false positive findings. Lastly, not all measurements were obtained for all patients at each time point. Most importantly, we obtained few blood samples at T2. Most centers did not routinely perform blood laboratory testing at the first outpatient follow-up. Some parents or guardians refused blood sampling for research purposes only, which underscores the importance of a patient perspective in study design. The limited availability of blood samples at this time point may have hampered the statistical power for analyses, including these measurements. Therefore, conclusions drawn from data that included biomarkers at T2 should be interpreted with caution. The limited sample size at T2 could also explain why relatively little variability within the dataset (8%) was explained by principal component 2. Despite these limitations, we present a comprehensive analysis of echocardiographic and serum biomarker profiles surrounding stages of the Fontan palliation.

## 5. Conclusions

In conclusion, ventricular performance may be temporarily decreased following PCPC surgery, but not following TCPC surgery. We characterized distinct clinical phenotypes via biomarkers analysis. One phenotype was governed by serum levels of, among others, ITGB1, GDF-15, and EGFR. These biomarkers relate to superoxide anion regulation. Clinically, this phenotype relates to early post-operative status. A second phenotype, governed by PLC, MB, and COL1A1 levels, related to the stage of Fontan circulation. These biomarkers are involved in the organization of the extracellular matrix and development of the cardiovascular system. This study provides insight into the recovery from potential injury related to PCPC and TCPC surgery and temporarily impaired ventricular function. We identified a biomarker profile that related to adaptation to the post-PCPC and post-TCPC circulation. These findings may aid in the development of new biomarkers for identifying patients at risk, or in the discovery of novel targets for therapy to prevent or ameliorate myocardial injury.

## Figures and Tables

**Table 1:** Patient characteristics.

	Total Study Population (*n* = 60)	PCPC Group (*n* = 32)	TCPC Group (*n* = 28)	*p*
Age (years)	1.1 [0.6–2.6]	0.6 [0.4–0.9]	2.7 [2.2–3.8]	**<0.001**
Male sex	35 (58%)	19 (59%)	16 (57%)	0.861
BSA (m2)	0.46 [0.36–0.57]	0.36 [0.34–0.39]	0.57 [0.53–0.64]	**<0.001**
Dominant ventricle				0.956
LV	22 (37%)	12 (38%)	10 (36%)	
RV	31 (52%)	16 (50%)	15 (54%)	
Indeterminate	7 (12%)	4 (13%)	3 (11%)	
Cardiac diagnosis				0.879
HLHS	17 (28%)	8 (25%)	9 (32%)	
TA	10 (17%)	6 (19%)	4 (14%)	
DORV	8 (13%)	4 (13%)	4 (14%)	
PA + IVS	5 (8%)	2 (6%)	3 (11%)	
Other	20 (33%)	12 (38%)	8 (29%)	
Extracardiac defects				0.419
-Heterotaxy syndrome	8 (13%)	6 (19%)	2 (7%)	
-Other	2 (3%)	1 (3%)	1 (4%)	
Previous surgeries	2 [1–3]	1 [1–2]	2 [2–3]	**0.001**
Previous catheter interventions	0 [0–1]	0 [0–1]	0 [0–1]	0.267
mBT		*n* = 18	*n* = 14	
Pulmonary artery banding		*n* = 10	*n* = 11	
Norwood procedure		*n* = 11	*n* = 9	
Rashkind		*n* = 7	*n* = 8	
Central shunt		*n* = 2	*n* = 2	
Ductal stenting		*n* = 2	*n* = 3	
PCPC		*n* = 0	*n* = 28	
Other		*n* = 9	*n* = 2	
Intervention time (min)	299 [261–356]	293 [256–311]	311 [281–348]	0.475
Perfusion time (min)	96 [64–115]	83 [57–113]	99 [90–119]	0.121
Crossclamp time (min)	47 [0–64]	0 [0–58]	59 [7–67]	**0.023**
Total hospital stay (days)	16 [10–27]	11 [7–38]	20 [14–25]	0.061
Total ICU stay (days)	2 [1–4]	2 [1–4]	2 [1–4]	0.549

PCPC: partial cavo-pulmonary connection; TCPC: total cavo-pulmonary connection; BSA: body surface area; LV: left ventricle; RV: right ventricle; HLHS: hypoplastic left heart syndrome; TA: tricuspid atresia; DORV: double outlet right ventricle; PA + IVS: pulmonary atresia with intact ventricular septum; mBT: modified Blalock(–Thomas)–Taussig shunt; ICU: intensive care unit. Statistically significant *p* values are displayed in bold.

**Table 2:** Echocardiographic and serum biomarkers at study time points. * Denotes a statistically significant difference between PCPC and TCPC (*p* < 0.05); ** *p* < 0.01; *** *p* < 0.001. Bold denotes a statistically significant *p* value.

	PCPC						TCPC					
	T1	T2	T3	*p* T1 vs. T2	*p* T2 vs. T3	*p* T1 vs. T3	T1	T2	T3	*p* T1 vs. T2	*p* T2 vs. T3	*p* T1 vs. T3
AV valve regurgitation	7	5	2	0.190	0.713	0.162	5	4	3	0.441	0.551	0.927
(Neo)aortic regurgitation	0	1	0	-	-	-	0	0	0	-	-	-
Systolic function												
SV GLS	−15.1 ± 5.6	−13.5 ± 5.2 *	−17.3 ± 4.5	**0.047**	**0.016**	0.357	−16.9 ± 4.6	−16.2 ± 3.8 *	−18.4 ± 3.0	0.246	0.106	0.218
SV lateral wall LS	−17.7 ± 5.8	−14.5 ± 4.9	−17.7 ± 5.0	**0.032**	0.105	0.946	−17.2 ± 3.9	−17.9 ± 4.2	−20.0 ± 3.7	0.906	0.348	0.169
TDI lateral AV S’ (cm/s)	6.6 ± 2.6	4.1 ± 1.4 **	5.1 ± 1.3	**0.004**	0.203	**0.043**	5.3 ± 1.6	5.3 ± 1.5 **	5.2 ± 1.1	0.617	0.301	0.434
AV-APSE (mm)	9.5 ± 3.8	7.1 ± 1.8 **	8.7 ± 0.59	0.051	0.105	0.425	9.0 ± 1.9	9.4 ± 2.6 **	8.6 ± 2.6	0.578	0.189	0.451
Diastolic function												
PW AV valve E (m/s)	1.0 ± 0.3 **	0.8 ± 0.2	0.7 ± 0.1	0.061	0.837	0.231	0.7 ± 0.2 **	0.7 ± 0.2	0.7 ± 0.2	0.930	**0.012**	0.051
PW AV valve A (m/s)	1.0 ± 0.4 **	0.7 ± 0.3	0.7 ± 0.2	**0.018**	0.469	0.089	0.7 ± 0.2 **	0.8 ± 0.2	0.6 ± 0.2	0.578	0.804	0.393
PW E/A ratio	1.0 [0.92–1.4]	1.0 [0.8–1.6]	0.9 [0.9–1.0]	0.109	0.109	0.999	1.0 [0.9–1.1]	0.9 [0.7–1.2]	0.9 [0.9–1.4]	0.424	0.734	0.547
Deceleration time (ms)	91 [84–103]	92 [69–131]	114 [96–129]	0.999	0.999	**0.039**	110 [96–128]	102 [79–109]	94 [79–113]	0.109	0.844	0.625
TDI lateral AV E’ (cm/s)	9.9 ± 3.9	6.5 ± 2.9	7.8 ± 3.6	**0.022**	0.250	0.096	8.5 ± 3.5	8.0 ± 4.0	8.9 ± 2.8	0.386	0.690	0.510
TDI lateral AV A’ (cm/s)	7.8 ± 3.3	5.1 ± 2.3	4.1 ± 1.4 **	**0.010**	0.803	**0.024**	6.1 ± 2.3	5.5 ± 1.7	6.7 ± 2.5 **	0.570	0.712	0.456
E/E’	8.9 [7.0–11.8]	11.0 [10.4–13.4]	9.3 [7.5–15.0]	0.240	0.313	0.844	8.6 [7.1–10.5]	9.0 [6.9–14.1]	7.9 [5.4–8.6]	0.569	0.129	0.688
Nt proBNP (pmol/L)	102 [36–124] ***	87 [62–153]	26 [17–46] *	0.901	**0.002**	**0.002**	23 [11–33] ***	49 [26–126]	15 [8–27] *	**<0.001**	**0.003**	0.495
Panel biomarkers principal component												
PC1	−0.3 ± 7.1	−2.2 ± 5.1	0.4 ± 5.9	0.606	0.133	0.745	2.5 ± 5.6	0.6 ± 6.2	−2.3 ± 3.4	0.174	0.460	**0.017**
PC2	2.0 ± 2.2 ***	7.8 ± 1.6 *	−1.0 ± 0.8 **	0.368	**0.045**	**<0.001**	−1.7 ± 0.9 ***	2.4 ± 2.8 *	−2.0 ± 1.0 **	**0.004**	**0.003**	0.529
PC3	2.4 ± 1.8 ***	−0.8 ± 0.1 *	−0.2 ± 1.8	0.636	0.245	**<0.001**	−0.0 ± 1.6 ***	−3.5 ± 2.9 *	−0.9 ± 2.1	**0.011**	**0.020**	0.222
PC4	−0.6 ± 2.4	−0.0 ± 0.2 *	−0.4 ± 1.7	0.434	0.349	0.840	−0.5 ± 2.4	2.1 ± 1.6 *	0.6 ± 2.3	0.079	0.237	**0.013**

PCPC: partial cavo-pulmonary connection; TCPC: total cavo-pulmonary connection; SV: single ventricular; GLS: global longitudinal strain; LS: longitudinal strain; TDI AV S’: tissue Doppler imaging atrioventricular valve S’; AV-APSE: atrioventricular valve annular plane systolic excursion; PW: pulsed-wave Doppler; AV: atrioventricular (flow); NT-proBNP: N-terminal pro-brain natriuretic peptide; PC: principal component.

**Table 3:** Principal components of the panel serum biomarkers. Conventional names and Uniprot codes can be found in the Supplementary Materials. Biological functions were determined with protein enrichment analysis.

	PC 1	PC 2	PC 3	PC 4
% of total variance explained	37%	8%	7%	5%
Contributing biomarkers	Urokinase receptor	ITGB1	PLC	JAM-A
	AXL	GDF-15	MB	CASP 3
	ICAM2	EGFR	COL1A1	GP6
	ALCAM	OPN	PDGF subunit A	PECAM 1
	TNF receptor 1	PON3	PAI	SELP
Biological functions	Cell surface	Regulation of superoxide anion generation	Extracellular matrix organization	Positive regulation of platelet activation
	Integral component of plasma membrane		Blood vessel development	Cellular extravasation
			Circulatory system development	Leukocyte cell–cell adhesion
				Leukocyte migration

**Table 4:** Expressions of individual serum biomarkers contributing to principal component 2 across study time points. Each individual biomarker differs at the early post-operative time point compared to other study time points.

	Total Cohort			PCPC			TCPC		
	T2	T1 and T3	*p*	T2	T1 and T3	*p*	T2	T1 and T3	*p*
ITGB1	4.6 ± 0.4	5.2 ± 0.7	**0.001**	4.2 ± 0.1	5.1 ± 0.9	**<0.001**	4.7 ± 0.4	5.4 ± 0.4	**0.002**
GDF-15	4.5 ± 0.8	3.9 ± 0.7	**0.048**	5.3 ± 1.2	3.9 ± 0.8	0.341	4.3 ± 0.6	3.8 ± 0.5	0.072
EGFR	1.9 ± 0.4	2.3 ± 0.6	**0.018**	1.9 ± 0.4	2.2 ± 0.7	0.487	1.9 ± 0.4	2.4 ± 0.4	**0.012**
OPN	9.7 ± 0.7	9.0 ± 0.9	**0.040**	10.3 ± 0.4	9.1 ± 1.1	0.063	9.5 ± 0.7	9.0 ± 0.5	0.133
PON3	3.9 ± 0.6	4.8 ± 0.9	**0.001**	4.2 ± 0.5	4.7 ± 1.0	0.420	3.8 ± 0.6	5.0 ± 0.7	**<0.001**

PCPC: partial cavo-pulmonary connection; TCPC: total cavo-pulmonary connection; ITGB1: integrin beta 1; GDF-15: growth/differentiation factor 15; EGFR: epidermal growth factor receptor; OPN: osteopontin; PON3: paraoxonase 3. Statistically significant *p* values are displayed in bold.

## Data Availability

Data are available from the authors upon a reasonable request.

## Supplementary Materials

The following supporting information can be downloaded at: https://www.mdpi.com/article/10.3390/jcdd10070289/s1, Table S1: Analyses for individual biomarkers.
