# Supplementary material for: Cardiac Function and Serum Biomarkers throughout Staged Fontan Palliation: A Prospective Observational Study
Source: J Cardiovasc Dev Dis. 2023 Jul 7;10(7):289. doi: 10.3390/jcdd10070289 (PMC10380373; doi:10.3390/jcdd10070289)
Supplement: Supplementary file 1 [file jcdd-10-00289-s001.zip › jcdd-2418131-supplementary.docx]

# Online supplement

## Principal component 1

Table S1: Analyses for individual biomarkers.

|  | ***Correlations between T1 levels and ICU stay for TCPC patients*** | |
| --- | --- | --- |
|  | ***r*** | ***p*** |
| Urokinase receptor | 0.48 | .098 |
| AXL | 0.43 | .138 |
| ICAM 2 | 0.49 | .090 |
| ALCAM | 0.42 | .153 |
| TNF receptor 1 | 0.58 | **.036** |

## Principal component 2

|  | ***Total cohort*** | | |  |  | ***PCPC*** | | |  |  | ***TCPC*** | | |
| --- | --- | --- | --- | --- | --- | --- | --- | --- | --- | --- | --- | --- | --- |
|  | ***T2*** | ***T1 & T3*** | ***p*** |  |  | ***T2*** | ***T1 & T3*** | ***p*** |  |  | ***T2*** | ***T1 & T3*** | ***p*** |
| ITGB1 | 4.6 ± 0.4 | 5.2 ± 0.7 | **.001** |  |  | 4.2 ± 0.1 | 5.1 ± 0.9 | **< .001** |  |  | 4.7 ± 0.4 | 5.4 ± 0.4 | **.002** |
| GDF-15 | 4.5 ± 0.8 | 3.9 ± 0.7 | **.048** |  |  | 5.3 ± 1.2 | 3.9 ± 0.8 | .341 |  |  | 4.3 ± 0.6 | 3.8 ± 0.5 | .072 |
| EGFR | 1.9 ± 0.4 | 2.3 ± 0.6 | **.018** |  |  | 1.9 ± 0.4 | 2.2 ± 0.7 | .487 |  |  | 1.9 ± 0.4 | 2.4 ± 0.4 | **.012** |
| OPN | 9.7 ± 0.7 | 9.0 ± 0.9 | **.040** |  |  | 10.3 ± 0.4 | 9.1 ± 1.1 | .063 |  |  | 9.5 ± 0.7 | 9.0 ± 0.5 | .133 |
| PON3 | 3.9 ± 0.6 | 4.8 ± 0.9 | **.001** |  |  | 4.2 ± 0.5 | 4.7 ± 1.0 | .420 |  |  | 3.8 ± 0.6 | 5.0 ± 0.7 | **< .001** |

## Principal component 3

|  | ***Pre PCPC*** | ***pre TCPC*** | ***post TCPC*** | ***p (ANOVA)*** |  | ***T1 levels for TCPC patients***  ***without complications*** | ***T1 levels for TCPC patients***  ***with complications*** | ***p*** |
| --- | --- | --- | --- | --- | --- | --- | --- | --- |
| PLC | 8.1 +- 0.4 | 7.5 +- 0.9 | 7.6 +- 0.4 | **.003** |  | 7.7 ± 0.3 | 7.3 ± 0.3 | .118 |
| MB | 5.1 +- 1.0 | 4.7 +- 0.8 | 4.6 +- 0.7 | .088 |  | 4.9 ± 0.6 | 4.5 ± 0.4 | .147 |
| COL1A1 | 4.3 +- 0.3 | 3.8 +- 0.5 | 3.7 +- 0.2 | **< .001** |  | 3.9 ± 0.2 | 3.6 ± 0.1 | **.015** |
| PDGF subunit A | 2.6 +- 1.1 | 2.8 +- 1.2 | 2.8 +- 1.0 | .465 |  | 2.1 ± 0.7 | 2.9 ± 0.9 | .096 |
| PAI | 5.0 +- 1.1 | 5.2 +- 1.4 | 5.4 +- 1.0 | .428 |  | 4.7 ± 0.9 | 5.4 ± 0.9 | .160 |

## Principal component 4

|  | ***Correlations between T1 levels and ICU stay for TCPC patients*** | |
| --- | --- | --- |
|  | ***r*** | ***p*** |
| JAM-A | -0.05 | .831 |
| CASP 3 | -0.06 | .805 |
| GP6 | -0.03 | .918 |
| PECAM 1 | -0.06 | .825 |
| SELP | -0.05 | .841 |
